# Substrate Recognition and Modification by the Nosiheptide Resistance Methyltransferase

**DOI:** 10.1371/journal.pone.0122972

**Published:** 2015-04-24

**Authors:** Sitao Yin, Hengyi Jiang, Dongrong Chen, Alastair I. H. Murchie

**Affiliations:** 1 Key Laboratory of Molecular Medicine, the Ministry of Education, Department of Biochemistry and Molecular Biology, Fudan University Shanghai Medical College, Shanghai 200032, PR China; 2 Institutes of Biomedical Sciences, Fudan University Shanghai Medical College, Shanghai 200032, PR China; St. Georges University of London, UNITED KINGDOM

## Abstract

**Background:**

The proliferation of antibiotic resistant pathogens is an increasing threat to the general public. Resistance may be conferred by a number of mechanisms including covalent or mutational modification of the antibiotic binding site, covalent modification of the drug, or the over-expression of efflux pumps. The nosiheptide resistance methyltransferase (NHR) confers resistance to the thiazole antibiotic nosiheptide in the nosiheptide producer organism Streptomyces actuosus through 2ʹO-methylation of 23S rRNA at the nucleotide A1067. Although the crystal structures of NHR and the closely related thiostrepton-resistance methyltransferase (TSR) in complex with the cofactor S-Adenosyl-L-methionine (SAM) are available, the principles behind NHR substrate recognition and catalysis remain unclear.

**Methodology/Principal Findings:**

We have analyzed the binding interactions between NHR and model 58 and 29 nucleotide substrate RNAs by gel electrophoresis mobility shift assays (EMSA) and fluorescence anisotropy. We show that the enzyme binds to RNA as a dimer. By constructing a hetero-dimer complex composed of one wild-type subunit and one inactive mutant NHR-R135A subunit, we show that only one functional subunit of the NHR homodimer is required for its enzymatic activity. Mutational analysis suggests that the interactions between neighbouring bases (G1068 and U1066) and A1067 have an important role in methyltransfer activity, such that the substitution of a deoxy sugar spacer (5ʹ) to the target nucleotide achieved near wild-type levels of methylation. A series of atomic substitutions at specific positions on the substrate adenine show that local base-base interactions between neighbouring bases are important for methylation.

**Conclusion/Significance:**

Taken together these data suggest that local base-base interactions play an important role in aligning the substrate 2’ hydroxyl group of A1067 for methyl group transfer. Methylation of nucleic acids is playing an increasingly important role in fundamental biological processes and we anticipate that the approach outlined in this manuscript may be useful for investigating other classes of nucleic acid methyltransferases.

## Introduction

The covalent modification of nucleic acids, proteins and small molecules through the addition of methyl groups (methylation) is an important part of cellular metabolism. Reversible methylation of DNA and histones introduces epigenetic modifications that can control cell lineage and cell fate [1–3]. Methylation at specific positions on the bases of DNA can alter the rate of sequence dependent DNA structural transitions [4,5] and abnormal patterns of DNA methylation are associated with a number of human diseases [6]. Ribosomal RNAs, tRNAs, mRNAs and long non-coding RNAs are also methylated. The *N* ^6^-methyladenosine (m^6^A) methylation of mRNA at internal adenosines [7,8] has been identified as a post transcriptional regulator of the circadian clock [9] and m^6^A methylation may also be reversed [10].

In most cases substrate molecules are methylated through methyl group transfer from the donor molecule S-adenosylmethionine (SAM) by methyltransferase enzymes. Alternatively, in some cases methyltetrahydrofolate and methyl-B12 may also serve as methyl group donors [11]. The basic chemistry of methylation by SAM–dependent methyltransferases normally proceeds by direct transfer of the methyl group from SAM to a nucleophillic substrate by an SN-2 like mechanism [12,13], although a class of SAM–dependent methyltransferases that methylate non-nucleophilic carbon atoms through an intermediate 5′-deoxyadenosyl 5′- radical has recently been identified [14].

Cellular rRNAs and tRNAs are extensively methylated by endogenous enzymes, the modifications are critical for efficient ribosome and tRNA function [15–17]. Resistance to antibiotics that target the bacterial ribosome is often conferred by methylation at specific rRNA nucleotides. The nucleotides that are methylated are located at the site of antibiotic interaction [18,19]. The antibiotics interact with key functional sites within the ribosome, and interfere with essential steps in protein synthesis. The main point of contact for the antibiotics within the ribosome is the rRNA.

Nosiheptide and thiostrepton are thiazole antibiotics that interfere with elongation factor G (EF-G) function by binding at a particular ribosomal site close to A1067 in 23S rRNA [20–22]. EF-G is the GTPase that promotes ribosomal translocation through GTP hydrolysis [23–25]. The resistance mechanism employed by the nosiheptide and thiostrepton producer organisms *Streptomyces actuosis* and *Streptomyces azureus* to protect endogenous ribosomes from drug binding is 2′O-methylation of position A1067 in 23S rRNA [25–27]. This region of 23S rRNA is also the binding site for the ribosomal protein L11 [28,29]. Crystal structures have been solved of this RNA in complex with L11 protein [27] or its C-terminal domain [30]. They reveal a complex tertiary fold in which residues A1067 and A1095 are close together. NMR structural analysis of this RNA in the presence of thiostrepton, shows that it binds in the vicinity of the 2′O position of A1067 [31,32]. Structures of whole ribosomes with thiostrepton and nosiheptide bound, show that they bind about 3Ǻ from the 2′O position of A1067 [33] and thus methylation at A1067 blocks thiazole binding directly [32]. The RNA is an efficient substrate for the nosiheptide (NHR) and thiostrepton (TSR) resistance methyltransferases *in vitro* [32,34–36].

**Fig 1:**
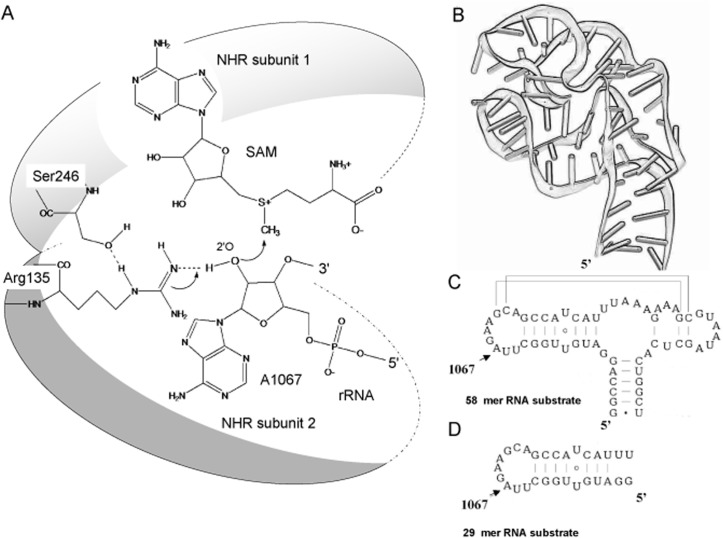
Substrate binding and modification by NHR. **A.** The proposed mechanism of methyl group transfer for NHR [36]. **B.** The substrate RNA is a small independently folding domain of 23S rRNA that adopts a complex tertiary fold [27,30]. **C.** The secondary structure of the RNA. The 58 nucleotide (nt) substrate for methylation, a series of mutations were made at A1067, the nucleotide is indicated by the arrow. **D.** The shortened 29 nt hairpin substrate, with A1067 marked.

Both NHR and TSR resistance methyltransferases display similar specificities for a series of fragment and model rRNA substrates and share 74% sequence identity [35–37]. The crystal structures of NHR and TSR proteins in complex with SAM reveal them to have a similar tertiary fold. Similarities in the tertiary structures and shared amino acid sequence alignments, suggest that they belong to and have a common evolutionary origin to the ‘SPOUT’ (SpoU-TrmD) superfamily of tRNA and rRNA methyltransferases [38–40]. The ‘SPOUT’ methyltransferases include the SpoU class of tRNA and rRNA 2′-O-methyltransferases and the TrmD family of tRNA N^1^ (G37) methyltransferases [39,41,42]. NHR shares a common fold with the SpoU (TrmH) tRNA methyltransferases that methylates the 2′-OH of G18 in tRNA. Both SpoU and NHR are dimers. Comparison between the crystal structures of NHR combined with mutational analysis led to the proposal of a catalytic mechanism for NHR that is analogous to that of SpoU [36,39,43]. In the catalytic model, inter-subunit hydrogen bonding between conserved Arg and Ser residues in the C-terminal domain define the catalytic centre, and orient the methyl group of SAM for nucleophillic attack by the 2′-oxygen of A1067 and methyl transfer with the loss of S-adenosyl-L-homocysteine (SAH) by an SN-2 like mechanism [36] (Fig 1).

Despite considerable efforts, co-crystal structures of NHR or TSR in complex with substrate RNA have yet to be determined [35,36]. In the absence of an RNA-protein structure the principles behind RNA substrate recognition and the mechanism of methyl group transfer by NHR have not been clearly delineated. For example, from the crystal structures neither NHR nor TSR dimers can accommodate the substrate RNA in its tertiary folded form, suggesting that changes in the conformation of the substrate RNA or the enzyme are required for methylation to take place. A number of questions therefore remain unanswered about the way that the NHR methyltransferase recognizes its substrate RNA. In this paper we show that only one functional subunit of the NHR protein dimer is required for binding and methylation of RNA. Our data support the idea that NHR dimer binding to RNA causes a conformational change in the substrate RNA. We also show that the enzyme can bind model RNA substrates with comparable affinities but the specificity of methylation is conferred by the nature of the substrate nucleotide.

## Materials and Methods

### Protein Expression and Purification

The construction and over expression of N-terminal hexahistidine tagged (His-tag) NHR protein (pET-28a-NHR) from *S*.*actuosis* was described previously [36]. A similar strategy was used to generate thioredoxin tagged NHR protein (pET-32M-NHR). Mutant proteins (His-NHR-R135A) were generated using the QuickChange Lightning Site-directed Mutagenesis protocol (Stratagene), and the Flag and HA tags were added to Trx-His-NHR and His-NHR-R135A separately by PCR. All His-tagged NHR proteins were affinity purified using an Ni-NTA (GE Healthcare) affinity column eluted with 500 mM imidazole as described previously [36]. Eluted proteins were further purified by gel filtration and concentrated to ~3mg/mL as previously described for wild-type proteins [36].

### Preparation of NHR heterodimers

Equivalent molar ratios of Flag-Trx-His-NHR (Flag-NHR) and HA-His-NHR-R135A (HA-NHR-R135A) proteins were mixed and incubated at 4°C overnight. Anti-HA beads were added and the mixture was incubated at 4°C overnight. Unbound protein was removed through centrifugation at 2,000 rpm for 3 min, and the beads washed three times with buffer A (50mM Na_3_PO_4_, 200 mM NaCl, pH 8.0). After centrifugation the immuno-precipitated complex were eluted with 10 mM HA peptide. Anti-Flag beads were then added into the eluted protein, and the mixture incubated at 4°C overnight. Unbound protein was removed by centrifugation at 2,000 rpm for 3 min, and the beads washed three times with buffer A. The immuno-precipitated heterodimers were recovered by elution with 10 mM Flag peptide, for comparison wild-type protein was purified the same protocol in parallel. Heterodimer composition was confirmed by SDS-PAGE and Western blotting.

### Electrophoretic mobility shift assay conditions

RNA samples of 58 and 29 nt RNAs (50 μM final concentration) were annealed at 95°C for 5 min, and cooled to room temperature. RNA-protein complexes were prepared by mixing the RNA (6–10 μM) with NHR protein and incubating the mixture for 1 hour at 4°C in reaction buffer (25 mM Hepes-KOH, 25mM NH_4_Cl, 5 mM MgCl_2_ and 5 mM DTT, pH 8.0).

**Fig 2:**
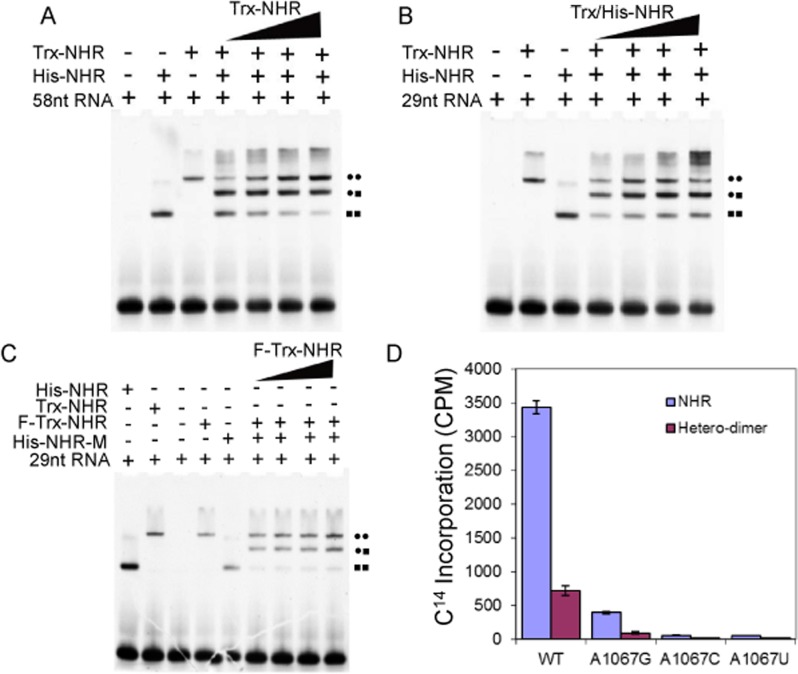
The stoichiometry of RNA binding by the NHR dimer. **A.** EMSA assays of 58 nt RNA binding by His-tagged NHR (His-NHR) or thioredoxin-tagged NHR (Trx-NHR). Tracks 1–3 (left to right), 10 μM 58nt RNA alone, 4 μM His-NHR and 4 μM Trx-NHR wild-type proteins separately; protein-RNA binding leads to a retardation in electrophoretic mobility compared to free RNA. Tracks 4–7, Trx-NHR was titrated against His-NHR by mixing at 4°C overnight, RNA-protein complexes were prepared by incubating the RNAs (10 μM) with 4 to 10 μM tagged-NHR protein (as indicated in the figure) for 1 hour at 4°C in reaction buffer. Formation of hetero dimers of NHR by His-NHR (short, S) and Trx-NHR (long, L) mixtures (4–10 μM) in the presence of 10 μM 58 nt RNA generates three distinct RNA-protein complexes corresponding to RNA-NHR(S)_2_ (■■), RNA-NHR(SL) (●■) and RNA-NHR(L)_2_ (●●). RNA-protein complexes were detected by staining with SYBR gold (Molecular Probes). **B.** EMSA assays of 29 nt RNA binding by His-NHR and/or Trx-NHR. Tracks 1–3 (left to right), 10 μM 29nt RNA alone, 4 μM His-NHR and 4 μM Trx-NHR wild-type proteins separately, protein-RNA binding leads to a retardation in electrophoretic mobility compared to free RNA. Tracks 4–7 (left to right) equivalent molar ratios of His-NHR and Trx-NHR were mixed at 4°C overnight. RNA-protein complexes were prepared by incubating the 29nt RNAs (10 μM) with 4 to 10 μM tagged-NHR (as indicated in the figure) for 1 hour at 4°C in reaction buffer. Formation of hetero dimers of NHR by mixing His-NHR (short, S) and Trx-NHR (long, L) in the presence of 29 nt RNA generates three distinct RNA-protein complexes corresponding to RNA-NHR(S)_2_ (■■), RNA-NHR(SL) (●■) and RNA-NHR(L)_2_ (●●). **C**. EMSA assays of 29 nt RNA binding by HA-His-NHR-R135A (His-NHR-M) mutant protein and/or Flag-Trx-NHR (F-Trx-NHR). Tracks 1–5 (left to right), His-NHR and Trx-NHR, 29 nt RNA alone, F-Trx-NHR and His-NHR-M proteins, separately, protein-RNA binding leads to retardation in electrophoretic mobility compared to free RNA. Tracks 6–9, F-Trx-NHR was titrated against His-NHR-M by mixing at 4°C overnight, RNA-protein complexes were prepared by mixing 6 μM RNA with 2 to 6 μM tagged-NHR proteins (as indicated in the figure) and incubating the mixture for 1 hour at 4°C in reaction buffer. Formation of hetero dimers of NHR by HA-His-NHR-R135A (short, S) and Flag-Trx-NHR (long, L) mixtures in the presence of 29 nt RNA generates three distinct RNA-protein complexes corresponding to RNA-NHR-M(S)_2_ (■■), RNA-NHR-M/RNA-NHR(SL) (●■) and RNA-NHR(L)_2_ (●●). **D.** Methyltransferase activity of the R135A-NHR heterodimer (HA-His-NHR-R135A/Flag-Trx-NHR). An NHR heterodimer comprising one wild-type subunit and one subunit containing the inactive R135A mutation was isolated by sequential affinity purification. The methyltransferase activity of R135-NHR heterodimer against wild-type, A1067G, A1067C and A1067U mutant RNAs in the presence of 1μCi ^14^C-AdoMet at 25°C from 30 min in 20 μL reaction buffer was assayed relative to concurrently purified NHR (Flag-Trx-NHR) protein (0.03 μM) against the same concentration (0.2 μM) substrate RNA molecules.

Differentially tagged and heterodimeric NHR protein samples at appropriate molar ratios as described in Fig 2 were mixed in reaction buffer and incubated at 4°C overnight prior to binding reactions.

For each sample RNA-protein complexes and free RNA were separated on a 6% polyacrylamide native gel for 1 h at 120 V and visualized by SYBR Gold staining (Invitrogen). The gels were scanned using a ChemiDocTM MP imaging system (Bio-Rad).

### RNA Preparation and Purification

The DNA templates containing the T7 RNA polymerase promoter sequence for transcription were obtained by gene synthesis. RNA was transcribed *in vitro* by T7 RNA polymerase (oligonucleotide sequences are listed in S1 Table). RNA was purified by gel electrophoresis in a 10% polyacrylamide 7M urea gel and RNA was collected by electroelution and recovered by ethanol precipitation, RNA concentrations were determined by Nanodrop spectrophotometer (ND-1000). D-spacer and modified 1-Me-A [44], Purine (as nebularine) and 7-deaza-A adenine nucleotides (Glen research) were incorporated into oligoribonucleotides by chemical synthesis with 2’O-silyl protection. The presence of modifications was confirmed by MALDI mass spectrometry.

### NHR *in vitro* Methylation Assay

NHR methyltransferase activity was measured as described previously [32,36], briefly RNAs at the appropriate concentrations were incubated in the presence of 0.05 μM NHR protein in the presence of [methy-^14^C] S-adenosyl methionine (SAM, AdoMet) (1 μCi) at 25°C for 30 minutes in 40 μL reaction buffer (25 mM Hepes-KOH, 25 mM NH_4_Cl, 5 mM MgCl_2_ and 5 mM DTT, pH 7.5). Reactions were stopped by the addition of 2% TFA, and the precipitate was filtered through a 96-well glass fiber filter plate. Incorporation of ^14^C was determined by liquid scintillation counting in a MicroBeta TriLux (Perkin Elmer 1450 LSC Luminescence Counter). Km, the Michaelis constant was calculated as previously described [32,36].

Mutant RNAs were assayed at an RNA concentration close to the Km for the RNA substrate (~200 nM for the 29 nucleotide RNA). The percentage relative specificity was quantified as the proportion of ^14^C incorporated into the substrate RNA as a percentage of the equivalent reaction by the wild type protein [26].

### The Anisotropy Assay

Transcribed RNA was labeled at the 3′ end with 5-fluorescein isothiocyanate (molecular probes) [45–47]. Labeled fluorescent RNA molecules were purified by denaturing gel electrophoresis, and recovered by electroelution and ethanol preciptitation. To characterize RNA and Protein interactions, fluorescence anisotropy measurements were performed in a Perkin Elmer LS55 luminescence spectrometer (excitation maximum (495nm), emission maximum (516nm)). Wild type protein (0–8 μM) was titrated in the presence of 2.5 μM RNA in 25 mM HEPES-KOH (pH 7.5), 25 mM NH_4_Cl, 5 mM MgCl_2_, 5 mM DTT [36] and the change in fluorescence anisotropy measured. The equilibrium dissociation constants (Kd) were determined by fitting to a one site binding model using the equation y = (B max*[Protein]/(Kd+[Protein]), in which B max is the change in anisotropy at protein saturation, [Protein] is the concentration of protein added, and Kd is the dissociation constant, as previously described [48,49].

## Results

### RNA recognition by the NHR dimer

**Fig 3:**
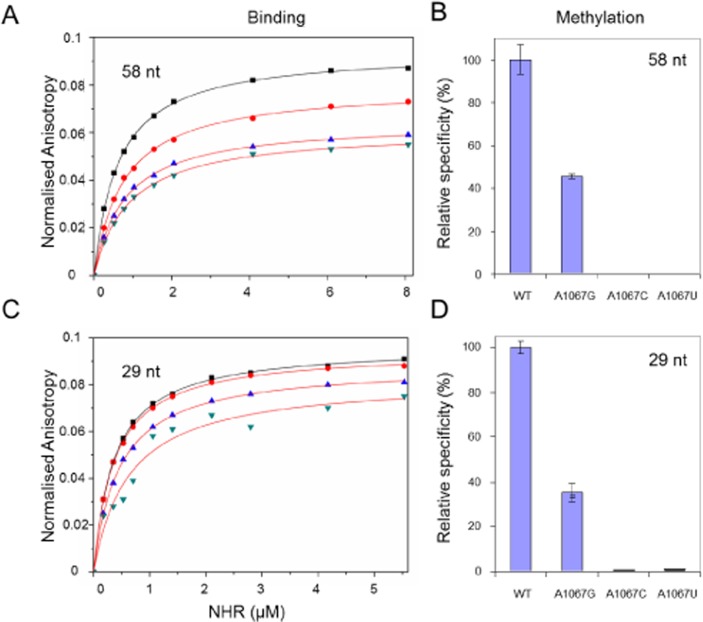
RNA binding and methylation by NHR. **A.** RNA binding by NHR protein measured by fluorescence anisotropy. Titration of NHR (1–8 μM) against RNA leads to a progressive increase in fluorescence anisotropy consistent with protein-RNA binding. Data represent the average of at least 3 independent measurements. Changes in fluorescence anisotropy with protein concentration were fitted to a one-site binding model and the dissociation constant (Kd) calculated. The change in normalized fluorescence anisotropy for mutations at A1067 of the 58 nt substrate; natural sequence A1067 RNA (closed squares, ■), A1067U (closed circles, ●), A1067C (close triangles, ▲) and A1067G (close inverted triangles, ▼). **B.** Relative specificity of methylation for mutations at A1067 of the 58 nt substrate. **C.** RNA binding by NHR protein measured by fluorescence anisotropy. Data represent the average of at least 3 independent measurements. The change in normalized fluorescence anisotropy for mutations at A1067 of the 29 nt substrate; natural sequence A1067 RNA (■), A1067U (●), A1067C (▲) and A1067G (▼). **D.** Relative specificity of methylation for mutations at A1067 of the 29 nt substrate.

Although the crystal structure of NHR has been solved [36], the principles behind RNA substrate recognition are not fully understood. NHR modifies 23S rRNA at adenosine A1067 which is located within an autonomously folding 58 nt region of rRNA which comprises helix 43 (H43) and H44 within domain II. Within this region the RNA adopts a complex tertiary structure and the equivalent position to A1067 was shown to be an efficient substrate for both the 58 nt RNA substrate and also the component 29 nt (H43) stem-loop [36] (Fig 1 and Fig 3).

The structures of both NHR and TSR in complex with SAM are dimeric and mutations to the interface between the dimer subunits are inactive or give low yields of soluble protein [36], suggesting that dimer formation stabilizes the active enzyme. RNA protein complexes of both enzymes migrate at high molecular weights in gel filtration and electrophoretic mobility shift assays [35–37], suggesting that the proteins may bind to the RNA as dimers. To investigate whether dimeric NHR is required for RNA binding, we prepared NHR protein containing two different N-terminal affinity tags; His-tagged NHR (His-NHR) and thioredoxin-tagged NHR (Trx-NHR). The 58 or 29 nt RNA substrates were prepared by *in vitro* transcription using T7 RNA polymerase (oligonucleotide sequences are listed in S1 Table). Electrophoretic mobility shift assays (EMSA) of 58 or 29 nt RNA substrates show that binding by His-NHR or Trx-NHR caused a significant retardation to the RNA protein complexes in the gel relative to unbound RNA, and that the RNA-His-NHR and RNA-Trx-NHR complexes can be resolved in the gel system in proportion to the relative sizes of each affinity tag (Fig 2). EMSA analysis of the 58 nt or 29 nt RNA substrate binding with heterodimers (His-NHR (short, S, ■) and Trx-NHR (long, L, ●)) reveals three distinct RNA-protein complexes corresponding to RNA-NHR(S)_2_ (■■), RNA-NHR(SL) (●■) and RNA-NHR(L)_2_ (●●) (Fig 2), confirming unambiguously that the NHR dimer is required for RNA recognition and binding (Fig 2). We also note the presence at higher protein concentrations of additional species of higher molecular weight corresponding to tetrameric RNA-protein complexes that have also been observed for the related methyltransferases TSR and SpoU [35,39].

### The NHR dimer requires only one functional subunit for RNA recognition and enzymatic activity

We have previously identified Arg135 as a candidate for the catalytic residue of NHR; mutation at this position causes a loss of methyltransferase activity [36]. To examine whether the NHR homodimer is important for RNA methylation, we constructed a heterodimer of NHR composed of one wild-type subunit and one subunit in which Arg135 was mutated to Ala and compared its RNA binding with the wild-type NHR homodimer using 29 nt wild-type RNA by EMSA. Heterodimers composed of the wild-type NHR were labelled with the Flag tag (Flag-NHR) and the inactive Arg135Ala mutant with the HA tag (HA-R135A-NHR) (His-NHR-M in the figure) were prepared as described in the previous section and analysed by EMSA with the 29 nt wild-type substrate RNA. EMSA analysis revealed three distinct RNA-protein complexes corresponding to the formation of hetero dimers of NHR by HA-His-NHR-R135A (short, S) and Flag-Trx-NHR (long, L) mixtures in the presence of 29 nt RNA generates three distinct RNA-protein complexes corresponding to RNA-NHR-M(S)_2_ (■■), RNA-NHR-M/RNA-NHR(SL) (●■) and RNA-NHR(L)_2_ (●●). Interestingly, the inactive R135A mutant homodimer (■■) binds to the RNA causing a shift in electrophoretic mobility, and the heterodimer composed of active and inactive subunits (●■) also binds the RNA efficiently. We also note that the inactive heterodimer complex binds the RNA more efficiently than the inactive mutant homodimer (Fig 2).

We also compared the methylation activity of the Arg135Ala NHR heterodimer with the wild-type NHR homodimer and a series of 29 nt wild-type and mutant RNA substrates. The heterodimer (Flag-NHR-HA-R135A-NHR) was purified through selective affinity purification by binding to Flag beads and followed by elution with Flag- peptide from a mixture of wild-type (Flag-NHR)_2_ and mutant (HA-R135A-NHR)_2_ NHR homodimers. The wild-type (Flag-NHR)_2_ homodimer was also purified in parallel using HA beads from the mixture. The wild-type homodimer purification serves as a control for the heterodimer purification process. The methyltransferase activity of purified heterodimer protein was then measured with a series of RNA substrates and compared to purified wild-type homodimer protein. Although the yield of heterodimer was rather low, we could compare the activity directly with the homodimer enzyme purified at the same time, under the same conditions in methyltransferase assays (Fig 2). Interestingly, the mutant NHR-R135A-NHR heterodimer showed consistent but reduced activity against the wild-type and mutant RNA substrates; in comparison the concurrently purified wild-type homodimer displayed normal activity against the wild-type RNA substrate and mutant RNAs (the mutant substrate RNA molecules are described in more detail in the following sections). From this we conclude that one functional subunit of the NHR dimer is required for methyltransferase activity.

### Binding and enzymatic activity of NHR with wild type and mutant 29nt or 58 nt RNA substrates

The NHR dimer can bind to both the 58 nt or 29 nt substrate RNAs. Both RNAs have been shown to be efficient substrates for methylation by the thiazole resistance methyltransferases [35,36]. Mutation of the A1067 position to U in the 58 nt RNA substrates has been shown to cause a reduction in methylation by the closely related thiostrepton resistance methyltransferase (TSR) [26]. Mutations to the 29 nt substrate at A1067 to G, C or U showed a significant reduction in methylation activity by NHR [36]. The enzymatic activity of NHR is dependent on two steps: the binding of the NHR to the RNA substrate and the catalytic process. To investigate why methylation of the mutant RNA substrate by NHR was significantly reduced, a fluorescence anisotropy-based binding assay was developed to measure the binding of NHR to a series of 29 and 58 nt RNAs that included the wild type sequence and the mutations G, C or U at A1067. This allowed the binding and methylation of the wild type and mutant RNA substrates to be compared. The wild type or mutant RNA substrates were prepared by *in vitro* transcription using T7 RNA polymerase and labeled with fluorescein [46]. NHR was expressed and purified as described previously [36]. In initial experiments RNA binding by NHR was characterized; the change in fluorescence anisotropy of fluorescein labeled 29 and 58 nt RNA molecules was measured on addition of NHR protein. In control experiments anisotropy measurements remained unchanged on titration of protein-free buffer (not shown). For the 58 nt or 29 nt RNA substrates; the wild type RNA (A1067) and the mutants (A1067G, A1067C and A1067U), titration of NHR protein leads to a progressive increase in fluorescence anisotropy consistent with protein-RNA binding (Fig 3). Changes in fluorescence anisotropy with protein concentration were fitted to a one-site binding model and the dissociation constant (K_d_) calculated. Interestingly NHR binds to the 58 nt or 29 nt RNA substrates with similar affinities in the nM range (Fig 3 and Table 1). Thus NHR does not distinguish between wild type and mutant RNAs at the level of RNA binding. The methylation assay shows that mutations at A1067 have a significant effect on the 58 nt or 29 nt RNA methylation such that the mutation A1067G shows 46% or 35% activity relative to the wild type RNA and methylation of the A1067C and A1067U mutants is almost completely abolished (Fig 3). From these data it is apparent that for the 58 nt or 29 nt substrates NHR do not distinguish between wild type and mutant RNAs at the level of RNA binding. However the nature of the nucleobase at 1067 has a significant effect on RNA methylation and the incorporation of a purine (A1067G) retains significant activity. Taken together this argues that substrate specificity of the enzyme is conferred by methyl group transfer (the catalytic step) to the 2′-OH of A1067 rather than at the level of RNA binding alone.

**Table 1:** Comparison of binding and methylation activity on model RNA substrates by NHR.

	Binding Kd (μM)	Methylation Km (μM)
29 nt RNA-wtA1067	0.37±0.03	0.20±0.03
29 nt RNA A1067G	0.37±0.03	0.24±0.04
29 nt RNA A1067C	0.46±0.04	N/A
29 nt RNA A1067U	0.63±0.08	N/A
58 nt RNA-wt A1067	0.62±0.01	0.38±0.05
58 nt RNA A1067G	0.77±0.02	0.5±0.03
58 nt RNA A1067C	0.80±0.02	N/A
58 nt RNA A1067U	0.91±0.02	N/A

Comparison of binding (Kd) and methylation activity (Km) on model 29 nt and 58 nt RNA substrates by NHR.

### Interactions between A1067 and neighbouring bases influence the enzymatic activity of NHR

**Fig 4:**
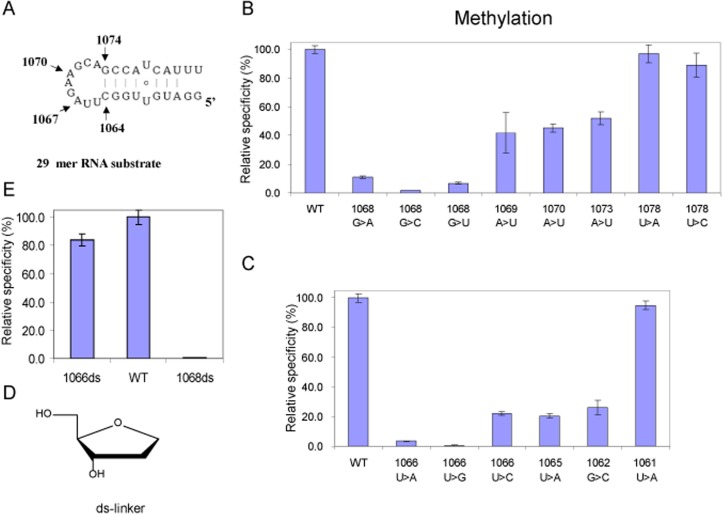
Investigation of neighbouring group influence on methylation of 29 nt substrate RNA. **A.** The 29 nt hairpin substrate, with A1067 and positions in the loop marked. **B.** The relative specificity for methylation of mutated nucleotides positioned 3′ to A1067. **C.** The relative specificity for methylation of mutated nucleotides located 5′ to A1067. **D.** Structure of the abasic deoxy (ds-linker) linker, that was used to replace U1066 (1066ds) and G1068 (1068ds). **E.** The relative specificity for methylation of linker modified 1066ds and 1068ds RNA.

For methyl group transfer NHR requires an adenine at the 1067 position of the RNA substrate. The methyl group from the donor SAM is transferred to the 2′-OH position of the ribose of A1067. Because the mutant A1067G displays significant but reduced activity and the pyrimidine mutants are inactive, we speculated that the conformation of the substrate nucleotide may influence the alignment of the 2′-OH towards methylation by SAM. To investigate the interaction between A1067 and the neighbouring bases, we made a series of mutant RNA substrates in which the neighbouring bases adjacent to A1067 were changed but A1067 remains unaltered. The effect of these mutants on the relative efficiency of methylation was measured in the presence of radiolabelled SAM and purified NHR. The mutations to the adjacent 3′ nucleotide; G1068 (G1068A, C and U) display a significant reduction in methyltransferase activity (Fig 4, summarised in Table 2), as do the mutations to the adjacent 5′ nucleotide U1066 (U1066A and G) (Fig 4 and Table 2) although interestingly the pyrimidine substitution U1066C retains 23% activity. Mutations to the 3′ outer loop nucleotides (A1069U, A1070U and A1073U) show intermediate levels of activity, and mutations within the 3′ stem have close to full activity (Fig 4 and Table 2). Mutations to the 5′ nucleotides (U1065A and U1062C) have lower activities and in the stem close to full activity (Fig 4 and Table 2). Taken together these data suggest that catalysis requires not only A1067 but also the neighbouring bases G1068 and U1066 and that interactions between A1067 and the neighbouring bases contribute to the methylation. Twenty-nine nucleotide oligoribonucleotides can be chemically synthesized with good efficiency; to further investigate the impact of the interactions between the adjacent bases on methylation at A1067 by NHR, the RNA substrate was chemically synthesized with the nucleotides U1066 or G1068 replaced by a deoxy-sugar linker i.e. without U or G (Fig 4 and Table 2). We then assayed the relative efficiency of methylation for these modifications (Fig 4 and Table 2). The substitution of the deoxy-sugar linker for G1068 effectively abolishes methylation by NHR suggesting that interactions between NHR, G1068 and A1067 have a critical role in orienting the 2′-OH for methylation by SAM (Fig 4 and Table 2). In contrast the introduction of the deoxy-sugar linker in place of U1066 achieved close to normal levels of methylation (Fig 4 and Table 2). From this we conclude that the presence of a flexible linker 5′ to the methyl acceptor can compensate for the loss of U1066, emphasising the important role that the positioning of the 2′-OH on the ribose has in methyl group transfer.

**Table 2:** The relative specificities for methylation of the 29 nt mutants.

No.	Sequence	Relative Specificity (+/-)	No.	Sequence change	Relative Specificity (+/-)
1	wt	100 (2.8)	18	1070 A>U	45.3 (2.8)
2	1067 A>G	35.4 (4.1)	19	1061 U>A	95.0 (2.7)
3	1067 A>C	0.7 (0.1)	20	1061 U>A, 1070 A>U	18.7 (0.8)
4	1067 A>U	0.6 (0.2)	21	1067A>U,1061U>A,1070A>U	0.26 (0.1)
5	1066 U>A	3.4 (0.3)	22	1065 U>A	20.4 (1.5)
6	1066 U>C	22.2 (1.5)	23	1073 A>U	52.0 (4.5)
7	1066 U>G	0.6 (0.2)	24	1065 U>A, 1073 A>U	89.3 (17)
8	1068 G>A	11.1 (1.0)	25	1062 G>C	26.2 (4.8)
9	1068 G>C	1.8 (0.2)	26	1078 U>A	96.9 (6.2)
10	1068 G>U	6.8 (0.8)	27	1078 U>C	89.0 (8.5)
11	1067 A>U,1066 U>A	2.26 (0.6)	28	U1066>ds	84.4 (5.9)
12	1067 A>U,1066 U>C	0.38 (0.1)	29	U1066>ds,A1067U	0.71 (0.2)
13	1067 A>U,1066 U>G	0.29 (0.02)	30	G1068>ds	0.45 (0.1)
14	1067 A>U, 1068G>A	0.53 (0.12)	31	A1067U,A1068>ds	0.32 (0.1)
15	1067 A>U, 1068G>C	0.36 (0.14)	32	A1067>1-Me-A	57.0 (2.2)
16	1067 A>U, 1068G>U	0.28 (0.01)	33	A1067>Purine	98.9 (2.5)
17	1069 A>U	42.0 (14.2)	34	A1067>7-Deaza-A	3.3 (0.5)

The relative specificity constants for methylation of the 29 nt mutants.

### Enzymatic activity of NHR on mutant RNA substrates in which A1067 is replaced by adenine structural analogues

**Fig 5:**
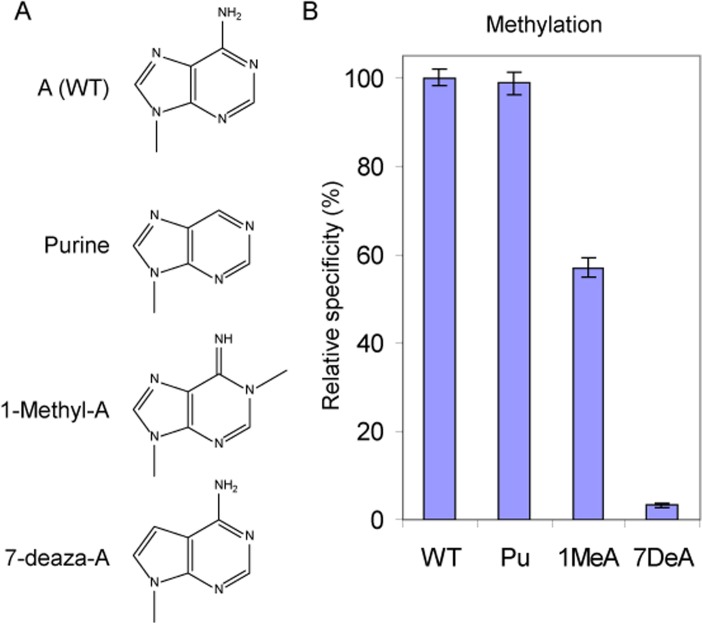
The effects of atomic substitutions to the substrate nucleotide on methylation of 29 nt substrate RNA. **A.** The nucleotide analogues used to investigate modification at A1067. **B.** The relative specificity for methylation of nucleotide analogues.

We have shown that the local environment around A1067 has a significant role in aligning the methyl group acceptor (the 2′-OH of A1067) for modification (Fig 4); we have also shown that the mutation A1067G retains significant activity (Fig 3). To further investigate the contribution of the base at A1067 towards methyltransferse activity, we introduced a series of atomic substitutions to the substrate adenine synthetically. Because A1067G shows a reduction in activity we focused initially on modifications to adenine. The 29 nt substrate RNAs were synthesized with 7-deaza-A and 1-methyl-A substitutions at A1067 (Fig 5) and methylation of these modified RNAs by NHR was measured as before. We observed that methylation of the 7-deaza-A (7-De-A) modified RNA substrate was reduced to background levels suggesting that interactions at N^7^ make an important contribution to methylation (Fig 5). In contrast the relative efficiency of methylation of 1-methyl-A (1-Me-A) was only reduced to 55% suggesting that methylation at N^1^ and the induced imino-tautomer at the exocyclic-6 position [50] are less viable for methylation (Fig 5 and Table 2). We next probed the contribution of the exocyclic amine by introducing purine (Pu) at A1067; significantly the relative efficiency of methylation of A1067Pu was indistinguishable from the wild-type RNA (Fig 5 and Table 2). This also confirms that local interactions at N^7^ are important for methylation. Taken together these data suggest that local base-base interactions play an important role in aligning the substrate 2’ hydroxyl group of A1067 for methyl transfer.

## Discussion

### RNA Recognition

NHR and TSR are members of the “SPOUT” (SpoUTrmD) super-family of RNA methyltansferases that appear to share a common evolutionary origin [51–54]. In common with the related methyltransferases SpoU and TrmD [39,41,42], inter-subunit hydrogen bonding between conserved amino acid residues contribute to dimerisation of NHR and TSR, cofactor binding and the formation of the catalytic centre that facilitates SAM methyl group transfer by an SN-2 mechanism [36,39–41,43,55] (Fig 1). Interestingly, for this class of enzymes, only the ribosomal pseudouridine methyltransferase Nep1 [56] and Trm5 the equivalent methyltransferase to TrmD for eukarya and archaea [57] that is active as a monomer and are the only members of this class of enzymes that have been co-crystalized with substrate RNAs [58].

Both NHR and TSR crystallize as dimers in complex with SAM, and have been proposed, based on EMSA and gel filtration analysis to recognize the substrate RNA as dimers [35,36]. Here we confirm unambiguously by comparative EMSA analysis of long and short heterodimers that NHR binds to the substrate RNA as a dimer. We also show that only one sub-unit of the dimer is required for methyltransferase activity. As antibiotic resistance methyltransferases, NHR and TSR differ functionally from the related tRNA methyltransferases TrmD and SpoU. Of these related RNA methyltransferases TrmD is responsible for the methylation of G37 at N^1^ in tRNA, a modification that is universally conserved, and essential for the fidelity of translation [52,59], while SpoU (TrmH) methylates the 2′-OH position of G18 in tRNA [60,61]. Compared to the tRNA methyltransferases SpoU, (TrmH) and TrmD [51,52], the natural substrate for NHR and TSR is the much larger 23S rRNA, and within this large RNA the enzyme methylates the specific nucleotide A1067. Nucleotide A1067 is positioned in a region of rRNA that folds to adopt a specific tertiary structure (Fig 1) [27,30]. Methylation by TSR is inhibited by the binding of ribosomal protein L11 or by antibiotic binding [32,34,62], implying that resistance to the thiazole antibiotics through the methylation of rRNA is conferred at the level of ribosome biogenesis, before the incorporation of L11 into the complete ribosomal subunit.

Both NHR and TSR enzymes can bind and methylate model RNA substrates; the 29 base hairpin loop and the 58 base L-11 binding RNA construct [35,36]. Significantly, compared to the larger RNA, the shorter 29 nt wild-type RNA had a higher affinity for the NHR enzyme and was a better substrate. In each case the (A1067) mutant RNA molecules, that were poor or inactive substrates for methylation, bound NHR protein with similar affinities (Table 1), suggesting that RNA binding affinity alone is not responsible for the selectivity of methyl group transfer by NHR protein. NHR does not therefore appear to distinguish between wild type and mutant RNAs at the level of RNA binding. Necessarily the potential tertiary interactions that stabilize the complex tertiary fold in the larger substrate RNA are absent from the shorter RNAs that are also better substrates. This is supported by comparison with a previous study on TSR in which certain mutations at positions that are buried in the core of the tertiary structure (A1069U, A1070U, and A1073U) of the 58 nt RNA were inactive [26], but with the shorter 29 nt RNA substrate, show intermediate levels of methylation (Fig 4). For the more exposed nucleotides methyltransferase activities are comparable between the 29 and 58 nt substrates (e.g. G1068U is inactive and U1078A is active). This is consistent with the idea that NHR binding induces a conformational change in the target RNA before specific methylation of the A1067 containing loop. Kuiper and Conn recently observed changes in the chemical and enzymatic footprints of TSR on the long substrate RNAs that are also consistent with a change in RNA structure upon TSR binding [37]. Such conformational changes to the RNA may be analogous to those observed with RumA and may be a feature of rRNA methyltransferases [63]. In contrast, the evolutionarily distinct methyltransferase Trm5, that has an analogous function to TrmD in eukarya and archaea is monomeric and requires structured, intact tRNA for methylation [57,58]. Interestingly, the dimeric enzymes TrmD and SpoU also recognize truncated RNA substrates [57,61,64,65]. This suggests that selective binding and manipulation of substrate RNA conformation by the enzyme dimers may be an important feature of RNA recognition by this family of enzymes.

The substitution of an abasic linker for the nucleotides at U1066 or G1068 had contrasting affects; the loss of the nucleobase at U1066 resulted in close to wild-type (84.4%) levels of methylation (Fig 4 and Table 2) whereas the loss of the base at G1068 effectively abolishes methylation by NHR. The substitution of A1067U combined with a 5′ or 3′ abasic linker (A1067U, U1066>ds) or (A1067U, G1068>ds) did not rescue methyltransferase activity (Table 2). Overall these data suggest that interactions between neighbouring bases contribute to the plasticity of the substrate RNA to position A1067 for methylation by SAM (Fig 4) such that interactions between G1068 and A1067 have a critical role in aligning the 2′-OH substrate for methyl group transfer.

Within the enzyme-RNA complex the microenvironment around A1067 also appears to make an important contribution to the orientation of the methyl group acceptor (the 2′-OH of A1067) for methylation. In addition, the nature of the substrate nucleobase at the 1067 position also makes a significant contribution to modification such that either pyrimidine (A1067U or A1067C) shows minimal levels of modification, whereas the purine A1067G shows intermediate levels of modification. To explore a possible role for purines substituted at A1067 in the positioning of the 2′-OH of A1067 (the methyl group acceptor) for methyl group transfer a series of atomic substitutions to the substrate adenine were introduced synthetically. The incorporation of guanosine analogues into tRNA model substrates has previously identified the importance of N^1^ and O^6^ of the substrate nucleotide G37 for TrmD methylation [66]. The data suggest that specific atoms positioned within the purine ring also play an important part in presenting the substrate 2′-OH for methyl group transfer. The N^7^ position of adenine appears to be essential for methylation; 7-deaza-A (7-De-A) modified RNA substrate was a poor substrate for NHR (Fig 5). In comparison the N^1^ and N^6^ positions of adenine are less important; methylation of N^1^ in 1-methyl-A (1-Me-A) also induces the imino-tautomer at the exocyclic-N6 position leading to intermediate activity (Fig 5). The N^6^ exocyclic amine in adenine appears to be dispensable for methylation; substitution of purine (A1067Pu) led to wild-type levels of modification (Fig 5). As a whole these data suggest that local base-base interactions play an important role in aligning the substrate 2’ hydroxyl group of A1067 for methyl group transfer.

## Supporting Information

S1 Table The RNA sequences used in this study. The sequences of the RNA oligonucleotides used in this study. Mutated nucleotides are indicated, and the positions of the mutations within the oligonucleotide are shown in red. NB the 29 nucleotide RNAs (numbers 28–34) that corporate the d-spacer or atomic substitutions were chemically synthesized, other RNA molecules were synthesized by *in vitro* transcription.
(DOC)
