# Supplementary material for: Substrate Recognition and Modification by the Nosiheptide Resistance Methyltransferase
Source: PLoS One. 2015 Apr 24;10(4):e0122972. doi: 10.1371/journal.pone.0122972 (PMC4409310; doi:10.1371/journal.pone.0122972)
Supplement: S1 Table — The sequences of the RNA oligonucleotides used in this study. Mutated nucleotides are indicated, and the positions of the mutations within the oligonucleotide are shown in red. NB the 29 nucleotide RNAs (numbers 28–34) that corporate the d-spacer or atomic substitutions were chemically synthesized, other RNA molecules were synthesized by in vitro transcription. (DOC) [file pone.0122972.s001.doc]

**Supporting information.**

**SI Table. The RNA sequences used in this study.**

| Number | name |  |
| --- | --- | --- |
| 1 | 29 wt-RNA | **G GAU GUU GGC UUA GAA GCA GCC AUC AUU U** |
| 2 | 29nt RNA 1067 A>G | **G GAU GUU GGC UUG GAA GCA GCC AUC AUU U** |
| 3 | 29nt RNA 1067 A>C | **G GAU GUU GGC UUC GAA GCA GCC AUC AUU U** |
| 4 | 29nt RNA 1067 A>U | **G GAU GUU GGC UUU GAA GCA GCC AUC AUU U** |
| 5 | 29nt RNA 1066 U>A | **G GAU GUU GGC UAA GAA GCA GCC AUC AUU U** |
| 6 | 29nt RNA 1066 U>C | **G GAU GUU GGC UCA GAA GCA GCC AUC AUU U** |
| 7 | 29nt RNA 1066 U>G | **G GAU GUU GGC UGA GAA GCA GCC AUC AUU U** |
| 8 | 29nt RNA 1068 G>A | **G GAU GUU GGC UUA AAA GCA GCC AUC AUU U** |
| 9 | 29nt RNA 1068 G>C | **G GAU GUU GGC UUA CAA GCA GCC AUC AUU U** |
| 10 | 29nt RNA 1068 G>U | **G GAU GUU GGC UUA UAA GCA GCC AUC AUU U** |
| 11 | 29nt RNA 1067A>U, 1066U>A | **G GAU GUU GGC UAUGAA GCA GCC AUC AUU U** |
| 12 | 29nt RNA 1067A>U, 1066U>C | **G GAU GUU GGC UCUGAA GCA GCC AUC AUU U** |
| 13 | 29nt RNA 1067A>U, 1066U>G | **G GAU GUU GGC UGUGAA GCA GCC AUC AUU U** |
| 14 | 29nt RNA 1067A>U, 1068G>A | **G GAU GUU GGC UUA AAA GCA GCC AUC AUU U** |
| 15 | 29nt RNA 1067A>U, 1068G>C | **G GAU GUU GGC UUA CAA GCA GCC AUC AUU U** |
| 16 | 29nt RNA 1067A>U, 1068G>U | **G GAU GUU GGC UUA UAA GCA GCC AUC AUU U** |
| 17 | 29nt RNA 1069A>U | **G GAU GUU GGC UUA GUA GCA GCC AUC AUU U** |
| 18 | 29nt RNA 1070A>U | **G GAU GUU GGC UUA GAU GCA GCC AUC AUU U** |
| 19 | 29nt RNA 1061 U>A | **G GAU GUA GGC UUA GAA GCA GCC AUC AUU U** |
| 20 | 29nt RNA 1061 U>A , 1070A>U | **G GAU GUA GGC UUA GAU GCA GCC AUC AUU U** |
| 21 | 29nt RNA 1067A>U, 1061 U>A , 1070A>U | **G GAU GUA GGC UUU GAU GCA GCC AUC AUU U** |
| 22 | 29nt RNA 1065U>A | **G GAU GUU GGC AUA GAA GCA GCC AUC AUU U** |
| 23 | 29nt RNA 1073A>U | **G GAU GUU GGC UUA GAA GCU GCC AUC AUU U** |
| 24 | 29nt RNA 1065U>A, 1073A>U | **G GAU GUU GGC AUA GAA GCU GCC AUC AUU U** |
| 25 | 29nt RNA 1062G>C | **G GAU GUU CGC UUA GAA GCA GCC AUC AUU U** |
| 26 | 29nt RNA 1078U>A | **G GAU GUU GGC UUA GAA GCA GCC AAC AUU U** |
| 27 | 29nt RNA 1078U>C | **G GAU GUU GGC UUA GAA GCA GCC ACC AUU U** |
| 28 | 29nt RNA 1066U>ds | **G GAU GUU GGC U-dspacer-A GAA GCA GCC AUC AUU U** |
| 29 | 29nt RNA 1066U>ds, 1067A>U | **G GAU GUU GGC U-dspacer-U GAA GCA GCC AUC AUU U** |
| 30 | 29nt RNA 1068G>ds | **G GAU GUU GGC UUA -dspacer-AA GCA GCC AUC AUU U** |
| 31 | 29nt RNA 1068U>ds, 1067A>U | **G GAU GUU GGC UUU -dspacer-AA GCA GCC AUC AUU U** |
| 32 | 29nt RNA 1067A>1-Me-A | **G GAU GUU GGC UU^1me^A GAA GCA GCC AUC AUU U** |
| 33 | 29nt RNA 1067A>Purine | **G GAU GUU GGC UU-purine- GAA GCA GCC AUC AUU U** |
| 34 | 29nt RNA 1067A>7-Deaza-A | **G GAU GUU GGC UU-7-Deaza-A- GAA GCA GCC AUC AUU U** |
| 35 | 58nt wt-RNA | **GGC CAG GAU GUU GGC UUA GAA GCA GCC AUC AUU UAA AGA AAG CGU AAU AGC UCA CUG GCU** |
| 36 | 58nt RNA 1067A>G | **GGC CAG GAU GUU GGC UUG GAA GCA GCC AUC AUU UAA AGA AAG CGU AAU AGC UCA CUG GCU** |
| 37 | 58nt RNA 1067A>C | **GGC CAG GAU GUU GGC UUC GAA GCA GCC AUC AUU UAA AGA AAG CGU AAU AGC UCA CUG GCU** |
| 38 | 58nt RNA 1067 A>U | **GGC CAG GAU GUU GGC UUU GAA GCA GCC AUC AUU UAA AGA AAG CGU AAU AGC UCA CUG GCU** |

The sequences of the RNA oligonucleotides used in this study. Mutated nucleotides are indicated, and the positions of the mutations within the oligonucleotide are shown in red. NB the 29 nucleotide RNAs (numbers 28-34) that corporate the d-spacer or atomic substitutions were chemically synthesized, other RNA molecules were synthesized by *in vitro* transcription.
